# A tutorial on the use of exploratory efficacy outcomes in uncontrolled phase I cell therapy trials

**DOI:** 10.1002/sctm.20-0116

**Published:** 2020-06-23

**Authors:** Jesse D. Troy

**Affiliations:** ^1^ The Marcus Center for Cellular Cures and Division of Blood and Marrow Transplantation, Department of Pediatrics Duke University School of Medicine Durham North Carolina USA

**Keywords:** biostatistics, clinical trials, phase I, patient‐reported outcome measures, research design, treatment outcome

## Abstract

Phase I cell therapy clinical trials evaluate the safety of novel biologic treatments and are often uncontrolled. Many of these studies also include exploratory efficacy outcome measures, which are frequently continuous measures of disease state or severity, or participant‐reported measures of symptom burden or quality of life. When such outcomes are included in uncontrolled phase I trials, they are typically serially assessed on the participants over time, and any improvement from baseline is interpreted as preliminary evidence of efficacy justifying a future, controlled trial. However, it is challenging to distinguish true improvement from regression to the mean in this design. The problem is exacerbated when trial entry criteria are based on extreme values of the outcome measure used to assess efficacy. It is possible to estimate the expected effect of regression to the mean when the natural history of the outcome measure is known, yet this is rarely done in practice. This article provides a refresher on regression to the mean for investigators designing early phase clinical trials in cell therapy and evaluates the potential for regression to the mean to have influenced conclusions drawn from recently conducted phase I cell therapy trials.

## Graphical abstract

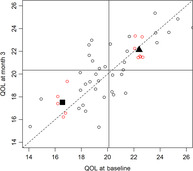

**Lessons learned**

• Regression to the mean is present in clinical trials whenever serial assessments of outcomes over time are not perfectly correlated.• Regression to the mean has a greater impact on results when trial entry criteria are based on having extreme values of the outcome measure at baseline.• The influence of regression to the mean in clinical trials can be avoided by use of a concurrent control group.

**Significance statement**

Interpretation of observed change in a continuous outcome measure as preliminary evidence of efficacy in the setting of an uncontrolled trial is likely to lead to erroneous conclusions due to the effect of regression to the mean. Evidence from single‐arm trials that assess efficacy through serial evaluation of continuous outcomes should not be used to make decisions about pursuing further research, or to select treatments for patients.

## 1 INTRODUCTION

This article is in the form of a statistical tutorial regarding the use of continuous outcome measures in phase I clinical trials that do not include concurrent or external controls. The objective of the tutorial is to communicate the statistical concern, namely, regression to the mean (RTM),^1^ that complicates efficacy assessment using the single arm trial design. The tutorial is designed to be accessible to anyone who has a basic familiarity with statistics.

The tutorial begins with a definition of RTM accompanied by an illustration using an example data set, followed by a demonstration of the ubiquitous nature of RTM and an explanation of how to estimate the impact of RTM on study results. An example application of estimating RTM is given using a hypothetical single arm trial of a cell therapy for multiple sclerosis (MS). Results of a literature review are then presented, assessing the potential impact of RTM on interpretation of recently published results in cell therapy clinical trials. Based on this review it is estimated that approximately one third of recently published early phase cell therapy trial results might exhibit RTM, possibly leading to erroneous conclusions regarding efficacy signals. Finally, the tutorial closes with some recommendations for early phase clinical trial design in cell therapy where assessment of continuous outcome measures is of scientific interest.

## 2 DEFINITION OF RTM

In a colloquial sense, RTM refers to the phenomenon in which patients who have initially high values tend to have lower values when measured again later.^1^ Likewise, patients who have initially low values tend to have higher values when measured subsequently. More generally, we can simply say that when serially assessing outcomes on participants, those who have extreme values at the first assessment will tend to have less extreme values at the second assessment.^2^ We can formalize this concept using a statistical model as follows.

Assume that we are interested in evaluating changes in quality of life (QOL) over time among patients who have been treated with an experimental cell therapy. Suppose that we have a valid and reliable tool to measure QOL and this tool provides a continuous, normally distributed outcome measure for which higher scores indicated better QOL. Let *Y*_*i*_ be the QOL score for a single subject at time *i* where the measurement time points are indexed from *i* = 1…*k*. The total number of measurements, *k*, and the spacing between measurements would be set based on the objectives of the study. At any time *i* the observed QOL is actually the sum of the subject's true QOL, *T*, plus some random error introduced by the measurement tool, *E*_*i*_, such that QOL for a single participant at time *i* is defined as *Y*_*i*_ = *T* + *E*_*i*_. Thus, *Y*_*i*_ is what the researcher observes, and although certain assumptions are made about *T* and *E*_*i*_, these variables are usually not observable.^3^

To finish specifying the model for QOL we must identify the sources of variation in the observed values, *Y*_*i*_. There are two sources of variation: (a) the natural variation between patients in QOL, regardless of the time point at which patients are measured, and (b) the variation among measures of QOL within the same patient over time. Patients' natural variation in QOL is accounted for by the distribution of *T*. We already stated that we will assume this variation is describable by a normal distribution. To make the example concrete we will assume *T* has a mean of 50 and SD of 5. Based on these assumptions we know that 95% of patients will have QOL scores in the range of 40 to 60. The second source of variation in the model—within‐patient variation—is represented by the distribution of *E*_*i*_, which has, by definition, mean 0 and, for our example, a SD of 5. Therefore, for a given person who has true QOL of 60, we would expect the distribution of their observed values, *Y*_*i*_, to be centered at the true value of 60 with 95% of their observed values falling between 50 and 70. To formalize the concept that *E*_*i*_ represents random noise in the measurement, we say that individual values of *E*_*i*_ are not correlated with each other, and values of *E*_*i*_ are also not correlated with the true value, *T*. ^3^

We can now understand RTM in light of this statistical model as shown in Table 1.^4^ Assume that in the population of patients we would treat there are 1 000 000 people with QOL at the mean value of 50. A total of 10 000 patients have QOL = 40, and 100 have QOL = 30. The distribution is symmetrical, so the same numbers of patients have true QOL above the mean at values of 60 and 70. Next, assume that 98% of patients have observed QOL with measurement error equal to 0. Furthermore, assume that 1% of observed QOL have measurement error equal to −10 points and that 1% have measurement error equal to +10 points.

**TABLE 1:** True vs observed values of a hypothetical quality of life (QOL) assessment illustrating regression to the mean

		Observed QOL				
Population, n	True QOL	*Y*_*i*_ = 30	*Y*_*i*_ = 40	*Y*_*i*_ = 50	*Y*_*i*_ = 60	*Y*_*i*_ = 70
100	*T* = 30	98	1	0	0	0
10 000	*T* = 40	100	9800	100	0	0
1 000 000	*T* = 50	0	10 000	980 000	10 000	0
10 000	*T* = 60	0	0	100	9800	100
100	*T* = 70	0	0	0	1	98

Reading across the rows in Table 1 we see that true values are at the center of the distribution of observed values, as expected. For example, among patients with true QOL of 60 (fourth row of Table 1; *T* = 60) the distribution of observed values is centered at *Y*_*i*_ = 60. In fact, if this were not the case, then the measurement tool would be seriously flawed! Reading down the columns of Table 1 shows something more interesting. For example, patients with observed values of 60 (*Y*_*i*_ = 60) have a range of true values between 50 and 70. However, many more of these patients have true QOL of 50 (10 000 patients) than have true QOL of 70 (1 patient). The simple reason for this is that there are so many more patients in the population with QOL values closer to the mean of 50 than there are who have true QOL values as extreme as 70. One can make a similar observation for patients with lower observed values as well, for example, *Y*_*i*_ = 40.

Now consider the repeated assessment of QOL on patients over two time points, *i* = {1, 2}. Then we have the following for any given subject: *Y*_1_ = *T* + *E*_1_ and *Y*_2_ = *T* + *E*_2_. Most importantly, under the assumptions of the model, *E*_2_ is not correlated with *E*_1_ or *T*. This implies that the expected value of the measurement error at the reassessment is 0 (just as it was at the first assessment) regardless of the amount of error present at the first assessment, or the underlying true value. To put it concretely, consider again patients with observed QOL of 60 at time *i* = 1. As explained above, nearly half of these patients have true values of 60 with the majority of the remaining patients having true values at the population mean of 50 and only a minority having more extreme values of 70. When these same patients are reassessed at time *i* = 2 we would again expect the observed values to be centered on the true value, in this case a true QOL of approximately 55. Thus, a group of participants with high observed values of QOL have somewhat lower values, which are closer to the mean, upon reassessment.

Although this statistical model establishes first principles for understanding RTM, the actual effect of RTM in a longitudinal study is somewhat more complex. This is the subject of the next section.

## 3 RTM IN AN EXAMPLE DATA SET

Suppose we conducted a single arm clinical trial in which we assessed a continuous, normally distributed QOL outcome measure on 50 patients. Again, we will assume that higher scores on this outcome measure represent better QOL. The first assessment was taken at entry into the trial, immediately prior to exposure to the experimental therapy. Assume for the sake of simplicity that there was only a single administration of the study product in this trial. The second assessment was taken later, at 3 months after the baseline assessment (the exact length of time is not important). Furthermore, suppose that QOL does not change over this period; that is, the distribution is identical—that is, the mean and SD are the same—at baseline and month 3 after treatment with the experimental therapy. Thus, the only fluctuation in the outcome measure over time is due to measurement error. In other words, our QOL assessment will produce a slightly different result each time we apply it to the same person, even though the person's QOL has not changed. Note that this scenario is equivalent to what would be a true null hypothesis in this design, that is, that the population of patients treated with the experimental therapy would not experience any change in QOL over a 3‐month period after exposure to the therapy.

**FIGURE 1:**
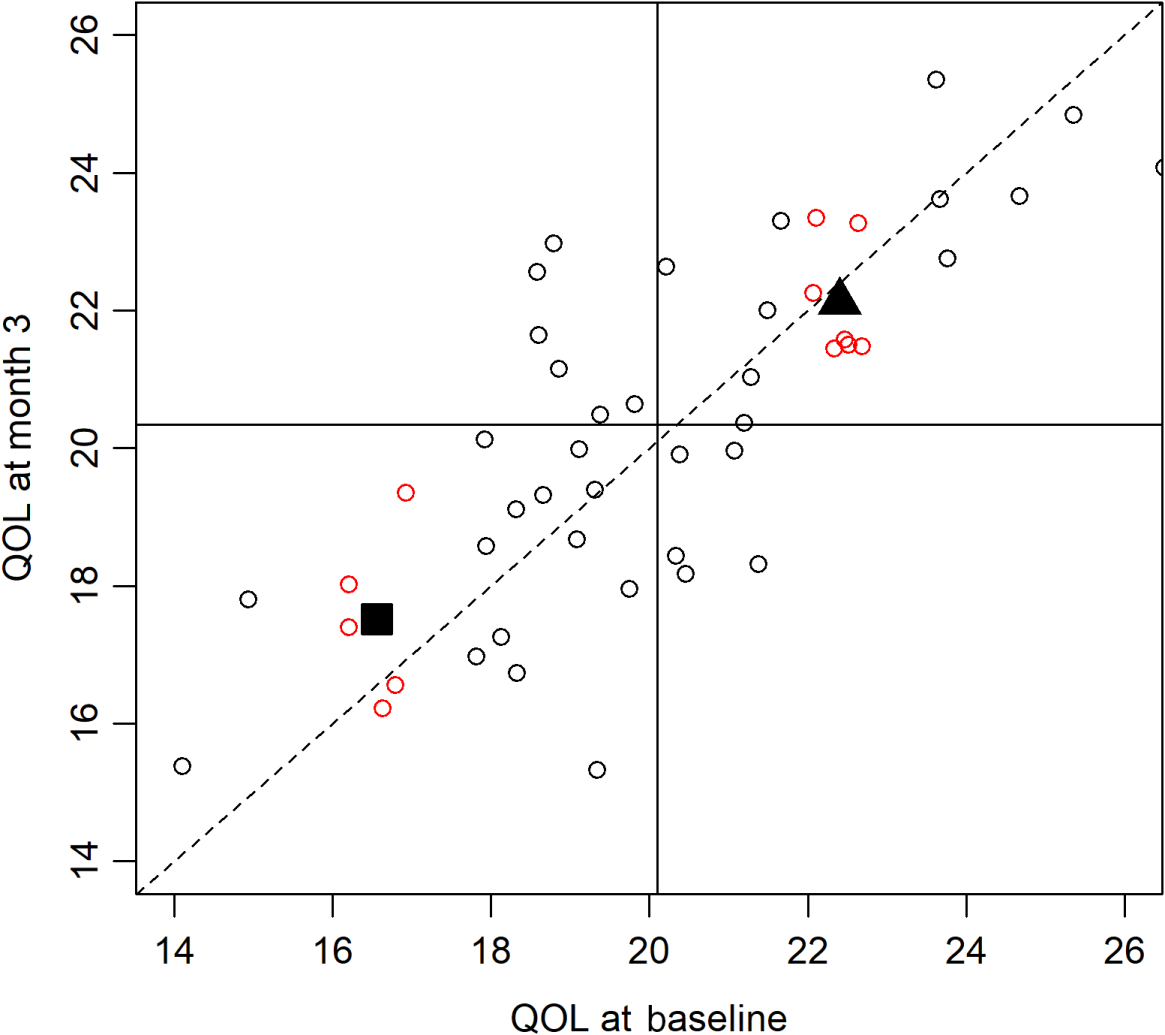
Example of regression to the mean in serial measures of quality of life (QOL). The plot represents results from a hypothetical single‐arm trial in which participants were followed up for 3 months postexposure to an experimental cell therapy. QOL was assessed at baseline (prior to exposure to the therapy) and again at month 3. Data for the example were generated from a bivariate normal distribution with mean of 20 and SD of 3 (for both components) and correlation of 0.8. Solid, perpendicular lines represent the cohort mean at baseline and month 3. The diagonal, dashed line is the line *Y* = *X* and reflects where the month 3 measurement would fall on the plot if there were perfect correlation between the baseline and month 3 measurements of QOL. Red dots in the bottom‐left quadrant are participants with baseline QOL in the range of 16 to 17 (with the black square showing the mean at month 3 for these participants), and the red dots in the top‐right quadrant are participants with baseline QOL in the range of 22 to 23 (with the dark triangle representing the mean at month 3 among this group). The proximity of the dark square (or triangle) to the solid line intersecting the *y*‐axis (the mean in the entire cohort at month 3) represents a regression toward the mean in these subgroups of participants who had extreme values of the outcome measure at baseline

Imagine that we obtain the results shown in Figure 1 from our hypothetical clinical trial. There are several things to note here. First, the solid perpendicular lines are drawn at the mean value of the baseline (*x*‐axis) and month 3 (*y*‐axis) measurements. As stated earlier, these distributions are assumed to be the same at the population level, and from the plot we can see the means of the sample at baseline and month 3 are essentially identical as expected (20.1 at baseline and 20.3 at month 3; program code for the example is available in the supplemental online data). The diagonal dashed line is the line *Y* = *X*, also called the line of agreement, which shows where the month 3 measure should appear on the plot if it were identical to the baseline measure. Because of measurement error, the two assessments are not the same for any given participant. Note also that points that fall on the line *Y* = *X* are roughly equidistant from the perpendicular lines that show the means at each time point.

The plot also shows an example of two groups of participants (the red dots). First, consider the group of participants who initially had low measures of QOL at baseline (in the range of 16‐17) shown in the lower left quadrant. The dark square indicates the mean of the month 3 measurement for this group of participants; that is, the *x* coordinate of the square is the mean of this subgroup at baseline (16.6), and the *y* coordinate of the square is the mean of this subgroup at the month 6 time point (17.5). Note that the dark square is positioned above the line of agreement. This indicates that the mean of the month 3 measurement for this group of participants is close to the mean of the month 3 assessment in the entire cohort. To be exact, the distance (on the *y*‐axis) from the square (17.5) to the mean at month 3 (20.3) is 2.8 points. Compare this to the distance from the dark square on the *x*‐axis (16.6) to the mean of the baseline measure in the entire cohort (20.1), which is 3.5. There are two things to note here. First, this group of participants had extreme low values of QOL at baseline that were far from the cohort mean at that time point. However, reassessment of these participants at month 3 shows that their mean QOL is now closer to the cohort mean than it was at baseline. In other words, these participants who had extreme values at the outset of the study have “regressed toward the mean” over time. A similar description could be given for the dark triangle in the upper right quadrant, which shows the mean for participants who have extreme high values of QOL (instead of low values) at the start of the study.

### 3.1 RTM is always reflected in imperfectly correlated serial measures

This example given above is not a special case. In fact, it is a scenario that is destined to occur whenever dealing with imperfectly correlated outcome measures. This can be understood easily through revisiting some basic concepts in linear regression analysis. We begin with some definitions as follows.

Let *X* = {*x*_1_, *x*_2_, …, *x*_*N*_} and *Y* = {*y*_1_, *y*_2_, …, *y*_*N*_} be the first and second assessment of a normally distributed outcome measure in a group of N participants followed over some arbitrary period of time. We can describe the relationship between the mean value of *Y* (the reassessment) and any given value of *X* (the initial assessment) using the familiar linear regression equation

Y=α+βX.

The regression equation describes a straight line through the data points (imagine the data arranged in a scatterplot such as that shown in Figure 1) where the *y* intercept, *α*, and the slope of the line, *β*, are determined such that a “best fitting line” is identified. A common method for estimating these parameters is the method of least squares, from which we know closed form solutions for the intercept and slope. One way of writing these equations is as follows^5^:

$$\hat{\beta }=r_{\mathrm{xy}}\frac{s_{y}}{s_{x}},$$

$$\alpha =\bar{y}-\hat{\beta }\bar{x},$$

where *r*_*xy*_ is Pearson's correlation coefficient; *s*_*y*_ and *s*_*x*_ are the sample SDs of the reassessment (*Y*) and initial assessment (*X*), respectively; and $\bar{y}$ and $\bar{x}$ are the sample means for each assessment. By substituting the above values into the regression equation and using algebra we can obtain the following representation of the linear regression equation:

$$\frac{y-\bar{y}}{s_{y}}=r_{\mathrm{xy}}\frac{x-\bar{x}}{s_{x}}.$$

This form of the regression equation articulates each observed value from *X* and *Y* (the initial and reassessments, respectively) in terms of the distance from the observed value to its mean, measured in units of SDs. In the case of perfect correlation, that is, ∣*r*_*xy*_∣  = 1, any observed value of *Y* (a postbaseline reassessment) is no closer to its mean than the corresponding value of *X* (the same participant's baseline assessment). Thus, when serial measures are perfectly correlated, there is no RTM. However, whenever there is imperfect correlation, that is, ∣*r*_*xy*_∣  < 1, then any given participant's reassessment (*Y*) is closer to its mean than the same participant's initial value (*X*) was to its mean. Therefore, when *X* and *Y* (initial and reassessment) are not perfectly correlated, there will always be RTM.

Unfortunately, the problem of RTM is not solved by expressing the outcome as a change from baseline. Using intuition based on the information presented thus far, one can imagine that participants who start the trial with extreme values will tend to have large amounts of change. The mathematics of the relationship between change and initial value are beyond the scope of the present article but are available elsewhere for interested readers.^6^

Finally, the effect of RTM can be quite large when trial entry criteria are based on extreme values of the outcome measure.^2^ This last point will become readily apparent in the next example, which demonstrates how to predict the amount of RTM that might be expected based on knowledge of the natural history of the outcome measure.

## 4 ESTIMATING EXPECTED RTM

There are some therapeutic areas in which the natural history of meaningful outcome measures is known. In these settings it is possible to estimate the amount of RTM that would be expected in the absence of any treatment effects.^7^ First, let *k* be the value of the outcome measure for which we are interested in estimating RTM. For example, in Figure 1 we evaluated RTM in participants with baseline QOL of *k* = 16. Next, we must know the mean, *μ*, and SD, *σ*, of the outcome measure in the population. These values could be obtained from a natural history study. Next, we can use this information to create a ratio that compares the probability of the value *k* in the population vs the probability of values greater than *k* in the population as follows:

$$a=\frac{\left(k-\mu \right)}{\sigma },$$

$$c=\frac{\phi \left(a\right)}{1-\Phi \left(a\right)}.$$

In this step we have defined a standard normal deviate, *a*, based on the cut point *k*. This enables us to create the ratio, *c*, comparing the frequency of *k* (calculated from the standard normal probability density function, *ϕ*) and the frequency of values above *k* (calculated using the standard normal cumulative distribution function, *Φ*). Lastly, we can also rely on the natural history data to provide an estimate of *ρ*_xy,_ the correlation between serial measures of the outcome (assumed to be constant; eg, first and second measures, third and fourth, or even first and fourth measures are assumed to be identically correlated). With this information at hand, it can be shown that the expected value of RTM in serial assessments of the outcome is.^7^

$$E\left[\mathrm{RTM}\right]=\mathrm{c\sigma}\left(1-\rho_{\mathrm{xy}}\right).$$

Similar to the regression example given previously, this formula illustrates that when serial measures are perfectly correlated, that is, ∣*ρ*_*xy*_∣  = 1, there is no RTM. Alternatively, RTM is largest when there is no correlation, that is, *ρ*_*xy*_ = 0. In addition, the formula also illustrates that RTM is partially dependent on the relative frequency of the selected cut point *k* in the population, that is, its percentile rank. For example, for *k* at the fifth percentile of a normally distributed population, *a* =  − 1.65 and *c* = 0.11. However, for values of *k* in the 95th percentile of the population, *a* = 1.65 and *c* = 2.1. Therefore, if trial eligibility is based on having extreme values of the outcome measure, then one might expect RTM to be prominent in the trial.

Unfortunately, the complete set of information required for the above calculations is seldom available in the literature. However, provided we can at least obtain information about the percentile rank of the cut point *k* in the population, we can use the data from our study to estimate *μ*, *σ*, and *ρ*_*xy*_, thus allowing us to use the above equation to estimate the expected amount of RTM. The details of this procedure are covered elsewhere and are not necessary to address for the purposes of this example.^7^ Finally, it should be noted that this method for estimating expected RTM is applicable specifically to normally distributed continuous outcomes. Although RTM may also manifest in ordinal or binary outcomes, some modifications to the above method may be required to evaluate the magnitude of RTM in those settings.

## 5 AN EXAMPLE USING A HYPOTHETICAL TRIAL TESTING A NOVEL CELL THERAPY FOR MS

MS is a disease for which the natural history of a clinically meaningful outcome measure has been documented. The Kurtzke Disability Scale (DSS) is an ordinal scale represented by the integers from 1 (least disabled) to 10 (most disabled), with each value on the scale having a detailed description of disability. A revised version of the scale, the Kurtzke Expanded DSS (EDSS), allows more detailed gradation of disability in increments of 0.5 instead of 1.^8^ An individual patient with Kurtzke EDSS less than 5 is said to experience a clinically meaningful change if his or her score is reduced by at least 1 point over time. Meaningful change for participants with Kurtzke EDSS ≥5 is defined as a reduction of at least 0.5 points.^9^

Let us now consider a hypothetical, single arm trial (n = 50) testing a single administration of an investigational cell therapy for MS. The primary outcome is the Kurtzke DSS, and the posttreatment follow‐up period is 6 months per participant. The inclusion criteria are specified such that the trial will sample from the population that is expected to experience the most benefit from the investigational therapy, indicated by a Kurtzke DSS score of 6 or higher. Based on an early natural history study of the Kurtzke DSS in patients with MS we know that 19% of the population has Kurzke DSS of 6 and 29% of the population has Kurtkze DSS greater than 6.^10^ Therefore, *c* = 0.19/0.29 = 0.66 (note that there is an implicit assumption here that the natural history study we refer to is an accurate reflection of the population we are sampling in our trial; investigators must be careful of this, especially in heterogenous disorders such as MS). Unfortunately, the natural history study did not publish estimates of *μ*, *σ*, and *ρ*_xy_, so we will rely on the data collected in our trial to estimate these parameters and calculate expected RTM using published formulas (we are not illustrating this step here). Suppose we have conducted the trial and obtained the results shown in Table 2.

**TABLE 2:** Example results illustrating regression to the mean in a hypothetical single arm trial of a cell therapy to treat multiple sclerosis: Mean change in Kurtzke Disability Scale from baseline to month 6

Parameter	Parameter value
Estimated *σ*	3.0
Estimated *ρ*_xy_	0.70
Observed mean at baseline, *X*	7.25
Observed mean at month 3, *Y*	6.75
Observed mean difference, |*Y* − *X*	0.50
Expected difference caused by regression to the mean	0.59

*Note*: The parameters *σ* and *ρ*_xy_ are the SD of the Kurtzke Disability Scale (DSS) in the hypothetical population that was sampled for this example clinical trial, and the correlation between repeated measures of the Kurtzke DSS, respectively. All parameters labeled “observed” are based on data collected during the trial. All parameter values were created for illustrative purposes only and are not based on any real cohort of patients with multiple sclerosis.

In this hypothetical example we obtained a result (mean difference of 0.5) that represents a clinically meaningful shift in this population. But can the observed change be attributed to the effect of treatment? By applying the methods discussed in the previous section we can see that the expected mean difference caused by RTM is 0.59 points. Thus, the observed change is within the limits of what we expect from RTM, and therefore it is unlikely that the experimental cell therapy has exerted a beneficial effect.

## 6 USE OF A CONTROL GROUP TO ADDRESS RTM

Based on the previous demonstrations it is clear that assessing efficacy through serial assessments of an outcome measure in a single group of clinical trial participants is difficult unless we have adequate knowledge from which we can calculate expected RTM for comparison with the observed results. In the absence of such knowledge a commonly accepted solution is to incorporate a control group into the study, preferably a randomized concurrent control (largely because such controls can be assured to be comparable with the treated participants with respect to factors prognostic for the outcome).^11^ The utility of the control group with respect to RTM is understood as follows. We have shown in this article that RTM is ever‐present, regardless of the treatment received. Thus, the control group represents the amount of RTM we expect in participants who do *not* receive the experimental cell therapy. The treated group represents both RTM *and* the effect of treatment (if the treatment in fact works). Thus, a difference in outcome between the treated participants and the controls excludes the effect of RTM. An added advantage of this approach is that identification of treatment effects using a control group is possible even when one does not know the natural history of the outcome measure in the population under study.

## 7 POTENTIAL IMPACT OF RTM IN PHASE I CELL THERAPY CLINICAL TRIALS

A literature search was conducted on 21 February 2020, to assess the potential impact of RTM on conclusions from recently published uncontrolled phase I cell therapy trials. A search query (Table S1) was executed in the PubMed database to identify phase I cell therapy clinical trials published in English from 1 January 2018 to 31 December 2019, that included the phrase “efficacy” or “treatment response” and did not test chimeric antigen receptor (CAR) T‐cell therapy (the primary outcomes of CAR T‐cell trials are typically binary or time‐to‐event outcomes, and the focus of this search was to identify studies that used continuous outcomes). A total of 26 articles met these inclusion criteria (Table S2). Articles were then excluded if they described the design of the trial and did not report results (2 articles), included a control group (2), or did not mention serial assessment of a continuous outcome measure in the abstract (13). The remaining nine studies that met all inclusion and exclusion criteria represented evaluation of novel therapies for erectile dysfunction, heart disease, spinal cord injury, autism, Crohn's disease, and osteoarthritis (one article each) and MS (three articles). All of these studies assessed at least one continuous outcome measure repeatedly on the participants over time, and two of the studies specified cut points in the continuous outcome measure as trial eligibility criteria. Based on this literature search, a total of 9 of 26 recently published early phase studies in cell therapy (34%) may have conducted preliminary efficacy evaluation without appropriate consideration for RTM. The practical impacts of this on product development are potentially large, possibly leading to subsequent phase II trials that do not demonstrate clear evidence of a treatment effect.

## 8 CONCLUSIONS AND RECOMMENDATIONS FOR EARLY PHASE CELL THERAPY TRIAL DESIGN

This tutorial demonstrated three statistical concepts that should be considered when deciding to use the single arm trial design for a phase I investigation of a cell therapy where continuous outcome measures will be used. First, RTM is nearly ubiquitous in serial assessments of continuous outcome measures, being present whenever serial measures are imperfectly correlated. Second, RTM is particularly problematic when trial entry criteria are based on the extreme values of the outcome. Third, in rare cases it is possible to estimate the amount of RTM that would be expected in the absence of treatment effects. However, the information required to do this is often not readily available. For these reasons, evaluation of efficacy based on serial assessment of continuous outcome measures in uncontrolled trials is not recommended.

It should also be noted that although RTM is an important motivating factor for understanding why efficacy is not estimable without a control, it is not the only motivating factor for using a control. For example, some single arm trial designs include simultaneous measurement of biomarkers and clinical outcomes that allow estimation of the correlation between biological changes and changes in the continuous efficacy outcome over time. Although this provides evidence that changes in biological activity are correlated with clinical outcome in treated patients, it does not provide any information as to how much *more* such biological activity or clinical outcome is modulated when the treatment was received as compared with when it was not (the treatment effect). Thus, in addition to serving as a solution to dealing with RTM, the use of a control group enables testing of scientific hypotheses that are not otherwise possible to test in a single arm trial.

The use of a concurrent, randomized control group remains the most accepted way to disentangle RTM from actual treatment effects. However, novel statistical designs, such as Bayesian dynamic borrowing, have recently emerged that leverage historical control data, potentially minimizing the number of contemporaneous controls required for a study.^12^ These designs may be beneficial for early phase cell therapy studies that incorporate efficacy assessments, especially in rare diseases in which the patient population is small, or in populations that express reluctance to consent to the possibility of assignment to a control.

In summary, this tutorial shows that interpretation of observed change in a continuous outcome measure as preliminary evidence of efficacy in the setting of an uncontrolled trial is likely to lead to erroneous conclusions. Therefore, evidence from single arm trials that assess efficacy through serial evaluation of continuous outcomes should not be used to make decisions about pursuing further research or to select treatments for patients.

## CONFLICT OF INTEREST

The author indicated no potential conflicts of interest.

## Supporting information


**Data S1.** Supporting information

## Data Availability

All the data generated or analyzed during this study are included in this published article.
