# Supplementary material for: A tutorial on the use of exploratory efficacy outcomes in uncontrolled phase I cell therapy trials
Source: Stem Cells Transl Med. 2020 Jun 23;9(11):1303–9. doi: 10.1002/sctm.20-0116 (PMC7581446; doi:10.1002/sctm.20-0116)
Supplement: Supplementary file 1 — Data S1. Supporting information [file SCT3-9-1303-s001.docx]

**Table S1: PubMed Search Query**

| **Date of Query:** | February 21, 2020 |
| --- | --- |
| **PubMed Query:** | "cell therapy"[All Fields] AND "Phase I"[All Fields] AND ("efficacy"[All Fields] OR "treatment response"[All Fields]) NOT ("chimeric antigen"[All Fields] OR "CAR-T"[All Fields]) AND (Clinical Trial[ptyp] AND English[lang]) AND (Clinical Trial[ptyp] AND "2018/01/01"[PDat] : "2019/12/31"[PDat] AND English[lang]) |

**Table S2: Literature Review Results**

| **First Author** | **Year** | **Title** | **Disease** | **Included?** | **Uncontrolled?** | **Serial Assessment of Continuous Outcome?** | **Outcome Used in Entry Criteria?** |
| --- | --- | --- | --- | --- | --- | --- | --- |
| Al Demour S, et al. | 2018 | Safety and Potential Therapeutic Effect of Two Intracavernous Autologous Bone Marrow Derived Mesenchymal Stem Cells injections in Diabetic Patients with Erectile Dysfunction: An Open Label Phase I Clinical Trial | Erectile Dysfunction | Yes | Yes | Yes | Yes |
| Bassetti B, et al. | 2018 | Linking cell function with perfusion: insights from the transcatheter delivery of bone marrow-derived CD133(+) cells in ischemic refractory cardiomyopathy trial (RECARDIO) | Heart/CVD/PVD | Yes | Yes | Yes | Yes |
| Bjorklund AT, et al. | 2018 | Complete Remission with Reduction of High-Risk Clones following Haploidentical NK-Cell Therapy against MDS and AML | Cancer | No |  | No |  |
| Bolli R, et al. | 2018 | Rationale and Design of the SENECA (StEm cell iNjECtion in cAncer survivors) Trial | Cancer | No - design paper | Not evaluated | Not evaluated | Not evaluated |
| Curtis E, et al. | 2018 | A First-in-Human, Phase I Study of Neural Stem Cell Transplantation for Chronic Spinal Cord Injury | Spinal Cord Injury | Yes | Yes | Yes | No |
| Fernandez-Aviles F, et al. | 2018 | Safety and Efficacy of Intracoronary Infusion of Allogeneic Human Cardiac Stem Cells in Patients With ST-Segment Elevation Myocardial Infarction and Left Ventricular Dysfunction | Heart/CVD/PVD | No | No | Not evaluated | Not evaluated |
| Gronhoj C, et al. | 2018 | Safety and Efficacy of Mesenchymal Stem Cells for Radiation-Induced Xerostomia: A Randomized, Placebo-Controlled Phase 1/2 Trial (MESRIX) | Cancer | No | No | Not evaluated | Not evaluated |
| Harris VK, et al. | 2018 | Phase I Trial of Intrathecal Mesenchymal Stem Cell-derived Neural Progenitors in Progressive Multiple Sclerosis | Multiple Sclerosis | Yes | Yes | Yes | Yes |
| Hijikata Y, et al. | 2018 | A phase I clinical trial of RNF43 peptide-related immune cell therapy combined with low-dose cyclophosphamide in patients with advanced solid tumors | Cancer | No | Yes | No | Not evaluated |
| Ishikawa T, et al. | 2018 | Phase I clinical trial of adoptive transfer of expanded natural killer cells in combination with IgG1 antibody in patients with gastric or colorectal cancer | Cancer | No | Yes | No | Not evaluated |
| Mathew JM, et al. | 2018 | A Phase I Clinical Trial with Ex Vivo Expanded Recipient Regulatory T cells in Living Donor Kidney Transplants | Solid Organ Transplant | No | Yes | No | Not evaluated |
| Mcgann PT, et al. | 2018 | Realizing effectiveness across continents with hydroxyurea: Enrollment and baseline characteristics of the multicenter REACH study in Sub-Saharan Africa |  | No - design paper |  |  | Not evaluated |
| Munakata W, et al. | 2019 | Phase I study of tirabrutinib (ONO-4059/GS-4059) in patients with relapsed or refractory B-cell malignancies in Japan | Cancer | No | Yes | No | Not evaluated |
| Murias M, et al. | 2018 | Electrophysiological Biomarkers Predict Clinical Improvement in an Open-Label Trial Assessing Efficacy of Autologous Umbilical Cord Blood for Treatment of Autism | Autism | Yes | Yes | Yes | No |
| Okonogi N, et al. | 2018 | Clinical outcomes of carbon ion radiotherapy with concurrent chemotherapy for locally advanced uterine cervical adenocarcinoma in a phase 1/2 clinical trial (Protocol 1001) | Cancer | No | Yes | No | Not evaluated |
| Okonogi N, et al. | 2019 | A Phase 1/2 Study of Carbon Ion Radiation Therapy With Concurrent Chemotherapy for Locally Advanced Uterine Cervical Squamous Cell Carcinoma (Protocol 1302) | Cancer | No | Yes | No | Not evaluated |
| Pender MP, et al. | 2018 | Epstein-Barr virus-specific T cell therapy for progressive multiple sclerosis | Multiple Sclerosis | Yes | Yes | Yes | Yes |
| Riordan NH, et al. | 2018 | Clinical feasibility of umbilical cord tissue-derived mesenchymal stem cells in the treatment of multiple sclerosis | Multiple Sclerosis | Yes | Yes | Yes | Yes |
| Serrero M, et al. | 2019 | Long-term Safety and Efficacy of Local Microinjection Combining Autologous Microfat and Adipose-Derived Stromal Vascular Fraction for the Treatment of Refractory Perianal Fistula in Crohn's Disease | Crohn's Disease | Yes | Yes | Yes | Yes |
| Song Y, et al. | 2018 | Human adipose-derived mesenchymal stem cells for osteoarthritis: a pilot study with long-term follow-up and repeated injections | Osteoarthritis | Yes | Yes | Yes | Yes |
| Subbiah V, et al. | 2018 | Cytokines Produced by Dendritic Cells Administered Intratumorally Correlate with Clinical Outcome in Patients with Diverse Cancers | Cancer | No | Yes | No | Not evaluated |
| Sundahl N, et al. | 2019 | Randomized Phase 1 Trial of Pembrolizumab with Sequential Versus Concomitant Stereotactic Body Radiotherapy in Metastatic Urothelial Carcinoma | Cancer | No | Yes | No | Not evaluated |
| Sung JA, et al. | 2018 | HIV-Specific, Ex Vivo Expanded T Cell Therapy: Feasibility, Safety, and Efficacy in ART-Suppressed HIV-Infected Individuals | HIV | No | Yes | No | Not evaluated |
| Vela M, et al. | 2018 | Haploidentical IL-15/41BBL activated and expanded natural killer cell infusion therapy after salvage chemotherapy in children with relapsed and refractory leukemia | Cancer | No | Yes | No | Not evaluated |
| Von Einem JC, et al. | 2019 | Treatment of advanced gastrointestinal cancer with genetically modified autologous mesenchymal stem cells: Results from the phase 1/2 TREAT-ME-1 trial | Cancer | No | Yes | No | Not evaluated |
| Yanagisawa R, et al. | 2018 | WT1-pulsed Dendritic Cell Vaccine Combined with Chemotherapy for Resected Pancreatic Cancer in a Phase I Study | Cancer | No | Yes | No | Not evaluated |

**Table S3. R Program Code for the Examples Given in the Tutorial**

## ---------------------------------------------------------------------------------------------

## rbvn()

## ---------------------------------------------------------------------------------------------

## Generate data from a bivariate normal distribution. Taken from:

## http://blog.revolutionanalytics.com/2016/08/simulating-form-the-bivariate-normal-

## distribution-in-r-1.html

## ---------------------------------------------------------------------------------------------

rbvn<-function (n, mu1, s1, mu2, s2, rho) {

X1 <- rnorm(n, mu1, s1)

X2 <- rnorm(n, mu2 + (s2/s1) * rho * (X1 - mu1), sqrt((1 - rho^2)*s2^2))

as.data.frame( cbind(X1, X2) )

}

#################################################################################################

# Generate data for the example of regression to the mean using the rbvn() function

#################################################################################################

#

# Note that the components of the bivariate normal distribution are identically distributed

#

set.seed(123)

example = rbvn(n=50,mu1=20,s1=3,mu2=20,s2=3,rho=0.8)

# X1 is the first assessment of QOL, X2 is the second assessment. The means and SD should be

# about the same. Any difference from each other (and from the population they are sampled from)

# would be due to sampling variability.

#

mean(example$X1)

sd(example$X1)

mean(example$X2)

sd(example$X2)

# Make a plot showing QOL at baseline on the X axis and QOL at Month 3 on the Y axis

# We'll highlight two groups: one that has low values at baseline (group1) and another

# that has high values at baseline (group2).

#

group1_lower = 16 # Greater than or equal to this value

group1_upper = 17 # But less than this value

group2_lower = 22 # Greater than or equal to this value

group2_upper = 23 # But less than this value

plot( example$X1,

example$X2,

xlab="QOL at Baseline",

ylab="QOL at Month 3",

xlim=c(14,26),

ylim=c(14,26),

col=ifelse( (example$X1 < group1_upper & example$X1 >= group1_lower) | (example$X1 >= group2_lower & example$X1 < group2_upper),"red","black"))

# Draw lines at the mean of the baseline and Month 3 measures

#

abline(v=mean(example$X1))

abline(h=mean(example$X2))

# Draw the line of agreement

#

abline(a=0,b=1,lty=2)

# Now look at the subset of participants with low and high values.

# What is the mean of the Baseline and Month 3 assessments for these participants?

#

m1_base = mean(subset(example$X1,(example$X1 < group1_upper & example$X1 >= group1_lower)))

m2_base = mean(subset(example$X1,(example$X1 < group2_upper & example$X1 >= group2_lower)))

m1_base

m2_base

m1 = mean(subset(example$X2,(example$X1 < group1_upper & example$X1 >= group1_lower)))

m2 = mean(subset(example$X2,(example$X1 < group2_upper & example$X1 >= group2_lower)))

m1

m2

# Label the means of these groups on the plot

#

points(x=m1_base,y=m1,bg="black",pch=22,cex=2)

points(x=m2_base,y=m2,bg="black",pch=17,cex=2)

# Calculate some distances

# First, for the square in the lower left quadrant:

# Distance on Y axis from the square to the cohort mean at Month 3

#

abs(m1-mean(example$X2))

# Distinace on the X axis from the square to the cohort mean at Baseline

#

abs(m1_base-mean(example$X1))

#

# Here are the analgous calculations for the triangle in the upper right quadrant

abs(m2-mean(example$X2))

abs(m2_base-mean(example$X1))

# Example calculations of the quantity c from the section titled Estimating Expected Regression to the Mean

dnorm(-1.65)

dnorm(1.65)

dnorm(-1.65)/(1-pnorm(-1.65))

dnorm(1.65)/(1-pnorm(1.65))
